# Single leg squat performance in physically and non-physically active individuals: a cross-sectional study

**DOI:** 10.1186/s12891-017-1660-8

**Published:** 2017-07-14

**Authors:** Silvia Gianola, Greta Castellini, Elena Stucovitz, Alice Nardo, Giuseppe Banfi

**Affiliations:** 1 Unit of Clinical Epidemiology, IRCCS Galeazzi Orthopedic Institute, Milan, Italy; 2 Center of Biostatistics for Clinical Epidemiology, School of Medicine and Surgery, University of Milano-Bicocca, Monza, Italy; 3 Department of Biomedical Sciences for Health, University of Milan, Milan, Italy; 4 Motion Analysis Laboratory, IRCCS Galeazzi Orthopedic Institute, Milan, Italy; 5 Scientific Directorate, IRCCS Galeazzi Orthopedic Institute, Milan, Italy; 6 Vita-Salute San Raffaele University, Milan, Italy

**Keywords:** Core stability, Clinical assessment, Lower extremity, Reliability of results, Reproducibility of results

## Abstract

**Background:**

Single-leg squat (SLS) is a functional test visually rated by clinicians for assessing lower limb function as a preventive injury strategy. SLS clinical rating is a qualitative evaluation and it does not count objective outcomes as kinematics data and surface electromyography (sEMG) assessment. Based on the SLS rating, the aims of this study were (i) to determine the clinical rating agreement among six raters and (ii) to assess kinematic and sEMG predictors of good SLS performance in physically and non-physically active individuals.

**Methods:**

Seventy-two healthy adults, divided in physically active and non-physically active groups, performed three SLSs on their dominant leg. Clinical ratings, kinematic data and sEMG were acquired. By using a validated clinical scale, six expert clinicians rated each SLS watching a video at three different time points. Intra and inter-rater agreement of clinical ratings were undertaken and a binary logistic regression analysis was used to determine kinematic and sEMG as predictors of SLS performance.

**Results:**

The weighted kappa coefficient for intra-rater reliability within each rater ranged between moderate and almost perfect agreement (0.55–0.85) whereas the weighted kappa coefficient for inter-rater reliability among raters was fair (0.34, time point 0; 0.31, time point 1; 0.30, time point 2). SLS analyses of physically active compared to non-physically active group showed a statistically significant difference in knee flexion and hip flexion (*p* = 0.041 and *p* = 0.023 respectively) and no difference in clinical ratings (*p* = 0.081). Greater knee flexion can predict the good SLS performance taking into account the belonging group (*p* = 0.019).

**Conclusions:**

Physically active individuals seemed to be at less risk to perform a non-good SLS and they had greater knee and hip flexions kinematics than non-physically active individuals. Knee flexion can predict the SLS performance quality therefore a greater knee flexion might also be considered a protective element from injuries.

**Trial registration:**

ClinicalTrials.gov identifier (trial has been registred retrospectively: NCT03203083. Date registration: June 21, 2017.

**Electronic supplementary material:**

The online version of this article (doi:10.1186/s12891-017-1660-8) contains supplementary material, which is available to authorized users.

## Background

Malalignment of lower extremity could affect the knee and hip kinematics during athletic movements [1, 2], e.g. causing injuries such as lesions of anterior cruciate ligaments [3], disorders as the iliotibial band friction syndrome [4] and the patellofemoral pain syndrome [5]. The single-leg squat (SLS) is a clinical functional test commonly used to evaluate clinical abnormal movement patterns of the lower limbs in terms of kinetic chain or co-ordinating muscle activity [2, 6, 7]. This scale accounts for the assessment of five dimensions: overall impression, trunk posture, pelvis in space, hip joint motion and knee join motion. The SLS is potentially promising as a functional test since it involves both daily activity and athletic task [8] and its validity and reliability have been examined by numerous researchers through different methods and assessment tools [2, 8–12]. Some authors tested the reliability of SLS using three-dimensional kinematics and subjective assessment [2, 9, 13–16]: for example, Crossley et al. proposed a clinical qualitative scale to evaluate the hip muscle function during the SLS [9]. SLS evaluation through this clinical scale can also be used to predict leg alignment and correlate it with hip and knee muscles function [12, 13, 17–19]. Functional activities are performed according to a kinetic chain, a coordinated sequence of motions of the body segments, which aims to achieve the desired task in the most efficient position, velocity and timing. Optimal activation of all body segments in the kinetic chain results in a high strength control with maximal performance and minimal risk of injury [20]. In several sports activities (e.g., running, throwing), the importance of the lower extremity alignment has been increasingly recognised [21]. However, no previous studies have sought to investigate whether the level of physical training and sport activities could affect the performance of the SLS.

We hypothesized that physically active individuals would be characterized by a more adequate kinematics (i.e coordinated sequence of actions of body segments) and more efficient muscle activity with better capability to perform a SLS task and fewer odds of being injured compared to non-physically active individuals. Moreover, we assessed the kinematic and the sEMG variables as predictors of the SLS clinical evaluation in physically and non-physically active groups not previously investigated in other studies.

## Aims

The aims of the present study were (i) to determine the intra- and inter-rater reliability for six clinicians when performing the SLS clinical rating assessment and to (ii) evaluate kinematic and sEMG predictors of the clinical evaluations for each group in order to recognise abnormal movement patterns and possible risks of injuries.

## Methods

### Study design

A cross-sectional study was performed to evaluate SLS performances in physically and non-physically active individuals. The study was approved by the San Raffaele Hospital’s Institutional Review Board (RC-L3017, 6–02-2014). Written informed consent was obtained from each participant. All research activities were conducted according to the Declaration of Helsinki. The reporting of the study followed the Strengthening the Reporting of Observational Studies in Epidemiology (STROBE) guideline [22].

### Sample size calculation

A previous study found a prevalence of “good performers” of SLS in 25% of the sample of healthy adults [9]. We hypothesized that a group of physically active individuals should have a higher prevalence of good performance, around 60%, with respect to a group of non-physically active individuals. The proportion of “good performers” in non-physically active individuals was assumed to be 25% under the null hypothesis while in physically active individuals was assumed to be 60% as the alternative hypothesis. The two-sided Z-Test with un-pooled variance was used. The significance level of the test was 5%. Given these estimates, a sample size of 28 subjects for each group equal to 80% power, taking into account 15% of dropouts, was considered sufficient to detect a difference between groups of 35%. Sample size calculation was obtained through Power Analysis Sample Software [23].

### Participants

A cohort of healthy young subjects was recruited. Inclusion criteria were the following: body mass index (BMI) between 18.5–25.5, age between 18 and 35 years old, written informed consent to participate, no musculoskeletal pain or history of lower extremity injuries lasting more than three months. We defined as “physically active individuals” subjects who perform sports activities more than 6 h per week and “non-physically active individuals” subjects who perform less than 2 h per week of sport activities [24]. All test sessions were conducted within a 3 months period.

### SLS procedure

All subjects were assessed in the Motion Analysis Laboratory at IRCCS Galeazzi Orthopedic Institute of Milan. In order to minimize bias, the subjects received standard information through a video on how to perform the SLS task [25]. The participants were evaluated barefoot and in underwear. They were asked to perform the exercise using their dominant leg, determined as the leg with which the participant would kick a ball.

The SLS task was executed according to a previous study [25]. Specifically, every subject stood in front of a force platform with arms crossed over the chest, made one step with the dominant leg onto the force platform, brought the other leg straight forward squatted down on the dominant leg as far as possible in a slow and controlled manner maintaining balance, returned to standing position on the dominant leg and finally stepped back out of the force platform back to the initial position. The exercise lasted approximately five seconds: SLS time was standardized using a metronome synchronized with an examiner who advised the SLS phases by counting three seconds for the descend phase and two seconds for the ascend phase.

All participants repeated the test three times. The execution of the SLS was recorded through a digital video camera (AXIS 210A, Axis communication, Sweden) positioned in front of the subject at a distance of three meters and a height of one meter. Digital images were stored in a DVD for clinical rating assessment.

### SLS clinical rating

A group of six clinicians, specialized in musculoskeletal rehabilitation, participated in the reliability study establishing the consensus panel. Two out of six clinicians were more expert in evaluating subject’s sport performance through SLS in their daily clinical practice. The original clinical scale of Crossley was adopted [9]. The five dimensions, overall impression, trunk posture, pelvis motion, hip and knee joints were assessed to rate the performance of a SLS task in “good”, “fair” or “poor” according to the rationale of judgment explained in the original study [9]. Each assessor evaluated three times the video of the SLS performance for each participant. SLS test were performed three times per subject but only the final repetition of these three trials was selected for the rating by the panel of examiners. The corresponding video was selected as it was expected to be the most representative of the participant’ abilities. The video was then evaluated by the panel at three different time points: immediately after the recruitment (time 0), two weeks after the first evaluation (time 1), at least one month after the first evaluation (time 2). Each examiner independently rated the video and was blinded to group and subject identity. At the end of the assessment process, intra-rater reliability was calculated using the scores obtained by the same examiners at the three different time points, while inter-rater reliability was calculated taking into account the scores of the video among all the examiners.

### Kinematics and electromyography set up

Three-dimensional kinematic data were collected during SLS tasks using a six infrared cameras (sample rate of 100 Hz) optoelectronic system (SMART-D, BTS Bioengineering, Italy). One force platform (Kistler, Amherst, NY) integrated into the walkway allowed the acquisition of the reaction forces exchanged with the ground. The local coordinate system was placed over the platform with the x-axis vector oriented concordantly to the direction of the task execution. The Helen Hayes marker set was used, applying 22 retro-reflective passive markers on specific anatomical landmarks and the Davis biomechanical model was used during acquisition and elaboration steps to obtain lower limb angles [26]. Hip angles were calculated between pelvis and thigh markers, knee angles between the thigh and the tibia markers, while pelvic angles were obtained using the right and left anterior superior iliac spine markers. The following kinematic variables were considered during the single-leg squat: ipsilateral hip flexion in the lateral plane, hip internal rotation, hip adduction, pelvic obliquity, knee flexion (lateral plane) and medio-lateral displacement (frontal plane). Data were averaged across the three trials.

Two different phases of the required task were considered: *pre-SLS phase* and *SLS phase*, based on the knee flexion of the dominant leg. The *pre-SLS phase* was determined as the time period starting with the foot contact on the force platform and ending after the participant stood on the dominant leg for one second, just before knee flexion. The *SLS phase* was defined as the time period starting at the end of the *pre-SLS phase* and ending when the participant came straight back on the dominant leg after the SLS (knee completely extended after the task).

After proper skin preparation, surface electrodes (H124SG Covidien, Ireland) placed on both legs were used to evaluate the surface Electromyography (sEMG) activity of the following muscles: tensor fasciae latae, rectus femoris, adductor longus, gluteus maximus and transversus abdominis. Since maximum voluntary contraction data were not available, a dedicated protocol for sEMG signal processing was created using Smart-Analyzer Software (BTS Bioengineering, Italy) able to estimate the percentage of basal muscle activity exhibited in the pre-SLS phase, compared to the muscle activity in the SLS phase. Each sEMG signal was band-pass filtered (20–400 Hz) and rectified and the area under the linear envelope of the signal concerning the two phases was evaluated. The ratio between the signal area of the pre-SLS-phase sEMG and that of the SLS-phase sEMG was computed for each muscle.

### Statistical analysis

Descriptive statistics (means and standard deviations) were reported for subjects’ anthropometric data. Intra and inter-rater agreements of the ordinal scale measure were calculated using weighted kappa coefficients [27]. Since more than two raters were involved, a generalized kappa is the recommended approach for evaluating intra and inter-rater agreement [28]. Differently from the weighted kappa statistic that compares observed frequencies of agreement with those expected by chance, the generalized kappa represents a comparison of the proportion of possible rater agreements to the proportion of classification in each category [10]. For inter-rater agreement among the six raters, a generalized kappa coefficient for each time point was obtained. Inter-rater agreement was determined between each couple of raters considering a mean score from the three trials for each rater. Intra-rater agreement was calculated for each rater by comparing all three ratings.

Interpretation strength of agreement (k-values) were made according to the scale offered by Landis and Koch (i.e. <0.00 poor, 0–0.20 slight; 0.21–0.40 fair, 0.41–0.60 moderate, 0.61–0.80 substantial, 0.81–1.00 almost perfect) [29].

A Kolmogorov-Smirnoff goodness-of-fit test was performed to verify the normal distribution of data within all the variables (kinematic and sEMG variables), and descriptive statistics (mean and standard deviation) were calculated. Where variables presented non-normal distribution, nonparametric tests were accordingly used.

Mann-Whitney tests for independent data were used to compare variables (clinical rating, good/fair/poor; kinematics; sEMG) between physically active and non-physically active groups. Statistical significance was observed at *p*-value ≤0.05. Binary logistic regression analysis was used to determine predictors of SLS rating. The independent variables were: kinematics, sEMG and the category of the population group (physically active and non-physically active individuals). The last variable was accounted as a categorical variable (1 = physically active, 2 = non-physically active individuals) in order to be imputed in the model. SLS rating binary dependent variable was considered as *good* versus *non-good*. For the non-good category, we merged *fair* and *poor* rating just for the analysis of predictors.

Statistical analyses were performed using the Statistical Package for the Social Sciences (SPSS) version 14.0 for Windows (SPSS, Chicago, IL, USA) and Microsoft Excel 2007 with Analyse-it function.

## Results

### Participant characteristics

**Fig. 1:**
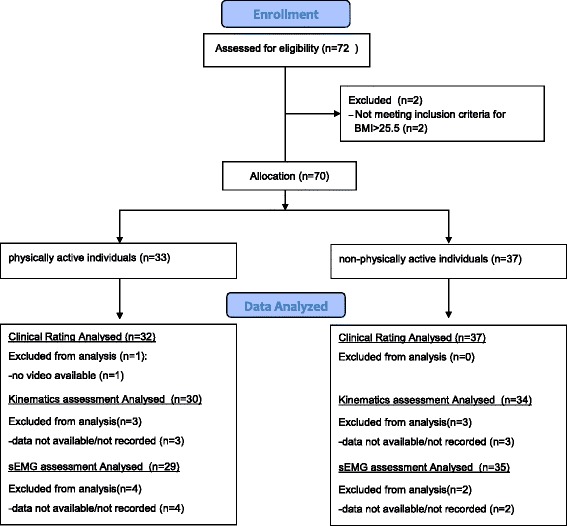
Trial flow diagram

Seventy-two individuals agreed to participate in the trial: 43 males and 29 females (25.1 ± 2.9 mean age, SD; 22.1 ± 2.1 mean BMI, SD). Thirty-five participants were allocated in physically active group and 37 in non-physically active group. Two physically active individuals were excluded because of a BMI slightly over the range considered. Trial participants flow diagram is reported in Fig. 1. In the end, the allocation consisted of 33 subjects (11 women and 21 men) and 37 subjects (17 women and 20 men) in physically and non-physically active groups, respectively. Eighty-three percent of the sample was right leg dominant (equally distributed in both groups). Baseline characteristics in the groups are shown in Table 1.

**Table 1:** Baseline characteristics of 70 subjects included

	Age (years)		Weight (kg)		Height (cm)		BMI (kg/cm^2)^		Gender (n)	
	Mean	sd	Mean	sd	mean	sd	Mean	Sd	Female	Male
Group physically-active (*n* = 33)	25.6	2.6	67.0	9.3	163.3	43.3	22.1	1.7	11.0	22.0
Group non-physically active (*n* = 37)	26.1	3.2	66.1	9.6	165.5	40.4	21.7	2.1	17.0	20.0

### Clinical ratings

#### Good, fair and poor performers

In the physically active group, 11 out of 32 participants (34.3%) were rated by the consensus panel as “good” performers, 19 (59.4%) as “fair” and 2 (6.25%) as “poor” performers. In the non-physically active group 7 out of 37 participants (18.9%) were rated as “good” performers, 24 (64.9%) as “fair” and 6 (16.2%) as “poor”. No statistically significant differences in clinical ratings were observed between physically active and non-physically active groups (See Additional file 1: Supplementary material – S1).

#### Reliability of clinical ratings agreements

The intra-rater comparison ranged from moderate to almost perfect strength of agreement (Generalized kappa: 0.55–0.85). The majority of weighted kappa of the intra-rater reliability exceeded the 0.75 substantial kappa of agreement (11 out of 18 rating trials) [30].

The inter-rater agreement was fair at each time point (i.e. time point 0: Generalized kappa 0.34, 95% IC 0.28–0.41). Inter-rater results for time point 1 and 2 and between each couple are summarized in Table 2.

**Table 2:** Intra and inter-rater agreement

	Weighted Kappa Coefficient	95% Confidence Interval		Standard Error
		Lower limit	Upper limit	
INTER-RATER RELIABILITY among six raters				
Generalized weighted kappa – Time point 0	0.34	0.28	0.41	0.04
Generalized weighted kappa – Time point 1	0.31	0.23	0.38	0.04
Generalized weighted kappa – Time point 2	0.30	0.23	0.37	0.04
INTER-RATER RELIABILITY between each couple of raters				
Rater 1 vs Rater 2	0.57	0.40	0.74	0.09
Rater 1 vs Rater 3	0.53	0.35	0.70	0.09
Rater 1 vs Rater 4	0.49	0.33	0.66	0.09
Rater 1 vs Rater 5	0.48	0.28	0.67	0.10
Rater 1 vs Rater 6	0.41	0.26	0.57	0.08
Rater 2 vs Rater 3	0.61	0.46	0.76	0.08
Rater 2 vs Rater 4	0.55	0.40	0.70	0.08
Rater 2 vs Rater 5	0.31	0.13	0.49	0.09
Rater 2 vs Rater 6	0.24	0.09	0.39	0.08
Rater 3 vs Rater 4	0.44	0.28	0.60	0.08
Rater 3 vs Rater 5	0.30	0.13	0.47	0.09
Rater 3 vs Rater 6	0.31	0.16	0.46	0.08
Rater 4 vs Rater 5	0.35	0.18	0.53	0.09
Rater 4 vs Rater 6	0.47	0.31	0.63	0.08
Rater 5 vs Rater 6	0.46	0.30	0.62	0.08
INTRA-RATER RELIABILITY				
Rater 1				
1 trial vs 2 trial	0.58	0.39	0.76	0.09
1 trial vs 3 trial	0.68	0.51	0.86	0.09
2 trial vs 3 trial	0.58	0.40	0.76	0.09
Generalized weighted kappa	0.61	0.51	0.72	0.05
Rater 2				
1 trial vs 2 trial	0.90^a^	0.81	0.98	0.04
1 trial vs 3 trial	0.85^a^	0.75	0.96	0.05
2 trial vs 3 trial	0.88^a^	0.78	0.97	0.05
Generalized weighted kappa	0.85^a^	0.79	0.92	0.03
Rater 3				
1 trial vs 2 trial	0.56	0.39	0.72	0.08
1 trial vs 3 trial	0.50	0.34	0.66	0.08
2 trial vs 3 trial	0.77^a^	0.64	0.90	0.07
Generalized weighted kappa	0.55	0.44	0.65	0.05
Rater 4				
1 trial vs 2 trial	0.77^a^	0.66	0.89	0.06
1 trial vs 3 trial	0.90^a^	0.82	0.99	0.04
2 trial vs 3 trial	0.83^a^	0.73	0.94	0.05
Generalized weighted kappa	0.80^a^	0.73	0.87	0.04
Rater 5				
1 trial vs 2 trial	0.73	0.58	0.77	0.07
1 trial vs 3 trial	0.71	0.57	0.86	0.07
2 trial vs 3 trial	0.78^a^	0.64	0.92	0.07
Generalized weighted kappa	0.70	0.61	0.79	0.05
Rater 6				
1 trial vs 2 trial	0.86^a^	0.78	0.95	0.05
1 trial vs 3 trial	0.81^a^	0.70	0.93	0.06
2 trial vs 3 trial	0.91^a^	0.82	1.00	0.05
Generalized weighted kappa	0.83^a^	0.77	0.90	0.03

The weighted kappa scores were interpreted as follows: 81% and higher. Excellent agreement; from 61% to 80%. substantial levels of agreement; from 41% to 60%. moderate agreement; and below 40%. poor to fair agreement. [data are taken from Landis and Koch. Landis JR. Koch GG. A one-way components of variance model for categorical data. Biometrics 1977]

^a^Interrater reliability of ordinal scale measures considered adequate for clinical use (≥0.75) [Portney and Watkins. 2009. Foundations of clinical research: Applications to practice. 3rd edn. Upper Saddle River. NJ. Pearson/Prentice Hall]

### Kinematics and sEMG predictors of SLS

Kinematic and sEMG data comparison between the two groups revealed no significant differences with the exception of knee flexion peak values (*p* = 0.041) and hip flexion peak values expressed in degrees (*p* = 0.023) which were greater in the physically active group than in the non-active one. Table 3 summarizes the results of the kinematic and sEMG muscle activation data.

**Table 3:** Two-independent samples test for kinematics and sEMG data in physically active and non- physically active groups

Variables	Group physically active		Group non-physically active		Mann-Whitney test	*p*-value
	Mean rank	Sum of rank	Mean rank	Sum of rank		
Kinematics (Unit of measure: degrees)						
Hip adduction	34.92	1047.50	31.36	1097.50	467.500	0.449
Hip internal rotation	32.85	985.50	33.13	1159.50	520.500	0.953
Hip flexion	38.15	1144.50	27.51	935.50	340.500	0.023*
Pelvic obliquity	33.22	996.50	31.87	1083.50	488.500	0.772
Knee flexion	38.17	1145.00	28.57	1000.00	370.000	0.041*
Knee medio-lateral displacement	33.28	998.50	32.76	1146.50	516.500	0.911
sEMG activation (Unit of measure: mV)						
Abductor sEMG	35.35	1060.50	30.99	1084.50	454.500	0.354
Gluteus Max sEMG	30.25	907.50	35.36	1237.50	442.500	0.278
Transversus sEMG	31.45	943.50	34.33	1201.50	478.500	0.541
Adductor sEMG	30.58	917.50	35.07	1227.50	452.500	0.340
Rectus femoris sEMG	31.68	950.50	34.13	1194.50	485.500	0.603

*p-value < 0.05

Subsequent analyses were performed on pooled data of the two groups. Table 4 shows the binary logistic regression analysis which identified the risk of the physically active individuals to be rated as non-good performers having each kinematic and sEMG variable as a predictor at a time. For example, as reported at the first listed variable in the table, i.e. hip adduction, we can assume that two subjects A and B have the same risk to be rated non-good (ODDS ratio = 1.069). If one subject (A) is physically active and the other is non-physically active (B) at the same amount of hip adduction angle, the subject B has an ODDS ratio of 0.327 of being a non-good performer. Indeed, the log-binomial regression showed that greater knee flexion can statistically significantly predict the good SLS performance assessed by the Crossley’s scale (*p* = 0.019).

**Table 4:** Binary logistic regression models for kinematic and sEMG predictors of SLS quality rated in physically active and non- physically active groups

	B	S.E.	Wald	Sig.	Exp(B)
Hip adduction					
kinematics	0.067	0.056	1.432	0.231	1.069
physically active and non- physically active groups	−1.118	0.607	3.397	0.065	0.327
Hip internal rotation					
kinematics	0.029	0.033	0.787	0.375	1.030
physically active and non- physically active groups	−1.032	0.591	3.045	0.081	0.356
Hip flexion					
kinematics	−0.001	0.023	0.001	0.977	0.999
physically active and non- physically active groups	−0.989	0.612	2.613	0.106	0.372
Pelvic obliquity					
kinematics	−0.036	0.052	0.483	0.487	0.964
physically active and non- physically active groups	−1.058	0.602	3.087	0.079	0.347
Knee Flexion					
kinematics	−0.064	0.027	5.490	0.019*	0.938
physically active and non- physically active groups	−0.598	0.632	0.896	0.344	0.550
Knee medio-lateral displacement					
kinematics	−0.033	0.037	0.823	0.364	0.967
physically active and non- physically active groups	−1.018	0.592	2.962	0.085	0.361
Abductor muscle					
sEMG	0.013	0.020	0.431	0.511	1.013
physically active and non- physically active groups	−1.071	0.594	3.257	0.071	0.343
Transversus muscle					
sEMG	0.001	0.017	0.001	0.972	1.001
physically active and non- physically active groups	−1.028	0.587	3.065	0.080	0.358
Gluteus Max muscles					
sEMG	0.024	0.029	0.670	0.413	1.024
physically active and non- physically active groups	−0.949	0.594	2.550	0.110	0.387
Adductors muscle					
sEMG	0.012	0.030	0.167	0.683	1.012
physically active and non- physically active groups	−0.991	0.593	2.789	0.095	0.371
Rectus femoris muscle					
sEMG	0.055	0.039	2.033	0.154	1.057
physically active and non- physically active groups	−0.968	0.599	2.612	0.106	0.380

B = coefficient; exp.(B) = estimated odds ratio

S.E. of B = Standard error of measurement

Wald = Wald statistic

Sig. = significance. *p < 0.05

## Discussion

In concordance with Crossley et al. results [9], high intra-rater and fair inter-rater agreements for the SLS clinical assessment were found. The majority of weighted kappa coefficients for the intra-rater reliability exceeded the value considered necessary for clinical use (≥0.75) [31]. Conversely, the weighted kappa for inter-raters indicated an agreement less favorable compared to the intra-reliability kappa coefficients. However, the inter-rater generalized kappa did not include the zero, indicating the presence of an agreement beyond what we expected. The number of possible scores (1 = good, 2 = fair, 3 = poor) and the numbers of raters (*n* = 6) may have influenced the results. An ordinal scale can be less accurate than a nominal scale (e.g. yes/no) due to the higher number of choices which produces an higher risk of disagreement [32]. Kappa values are also affected by distributions of disagreement. If one rater is more severe than another one, the resulting kappa value might be lower because the disagreement may be inflated not by causality but by orderliness [32]. It has been observed that in the clinical evaluation the two more experienced raters (rater 2 and rater 6) usually judged with a more systematic method proposing a possible not random disagreement (see Table 2).

Our results suggested that the rating into good, fair or poor categories (Crossley’s clinical scale) provided a reproducible clinical method to assess SLS performance. This scale is easier and faster to be used in the clinical setting compared to other clinical scales: it does not require any expensive equipment and allows immediate feedback to be given to the patient examined [33]. Despite the ambiguities in clinical judgements due to the subjective connotation, the Crossley’s scale provides a unique judgement accounting for all aspects needed to assess the lower limb alignment (overall impression, hip, knee, pelvis and ankle joints). This overall judgment would be a more reliable method compared to an evaluation based only on a specific body segment observation (e.g., only pelvis) [10]. Former authors have investigated rater’s agreement during lower extremity functional tasks such as the SLS, leading to a wide range of reliability values. Nevertheless, their findings cannot be directly compared to our current study due to methodological differences in study design [1]. Indeed, no other investigators have evaluated the reliability of an ordinal clinical scale for lower extremity alignment among such a large number of raters: the involvement of six raters can increase the strength of the external validity of our results.

A higher proportion of “good” performers (34.3%) was found in the physically active group compared to the non-physically active (18.9%) one, but differences in good, fair and poor ratings were not statistically significant. This could be mainly explained by the low frequency of sample distributed between the two groups and the wide range of practiced sport in the physically active group could even have flatten the differences in both groups.

Although findings showed no significant differences in clinical ratings between groups, kinematic data suggested higher knee and hip flexion peak values in favour of the physically active group. Precisely, knee flexion might be the strongest predictor of SLS quality performance between groups. We hypothesized that a deeper knee flexion might predict the good SLS performance: this suggests that having an adequate knee condition in terms of great muscle activity, coordination and proprioception might prevent injuries and thus, be a preventive factor. In concordance with our considerations, it has been showed in literature that a great knee flexion decreases thoracolumbar hyperextension in tennis players [34] and decreases anterior cruciate ligament loading [35]. Conversely, a decreased knee flexion has been associated to increased anterior cruciate ligament loading [35]. Besides, we showed an increased risk for non-physically active people (1/3) to be a non-good performers compared to the physically active ones in most of the kinematic and sEMG variables. It means that the non-physically active individuals might have three times more the risk to be rated as non-good performers compared to the physically active ones for almost all kinematic and sEMG variables. Although our findings were not statistically significant, we hypothesized that being physically active or non-physically active might modify the probability to be rated a good or non-good performer whereas the kinematic and sEMG variables might not predict the quality (good/non-good) of the performance with the exception of the knee flexion. Generally, physically active people seemed to have greater knee flexion values and less risk of non-good performance than non-active ones, suggesting that being physically active might be more protective for injuries thanks to the acquired quality of knee condition. According to Weeks et al. [2], our results highlighted the importance that clinicians should give to knee flexion in making their assessment. Therefore, the standardization of the depth of knee flexion task in rehabilitative exercises or during the clinical evaluation through the SLS could be set up as a suggestion for clinicians and end-users. The ability to detect knee and hip flexion angles during SLS test might be an important skill for clinicians to screen for leg malalignment, especially when clinicians account for athlete’s professional careers in injury prevention. However, other aspects should be investigated as the ability to detect valgus collapse, the trunk control or the early heel rise [7], which are also components of lower extremity injury risk not collected in our study.

In conclusion, we suggest exploring whether differences in sport specialties can change the performance of SLS through further studies.

Limitations of the study referred to the sEMG signal acquisition method and to the lack of lateral video registration. A frontal video data can not exactly reflect what usually happens in clinical practice but a later view of the exercise could have given a more comprehensive judgment. However, unidirectional video is used regularly. Furthermore, the Maximum Voluntary Contraction for each muscle was not available, so it was not possible to normalize the sEMG signal respect to the muscle maximum activity. Despite these limitations, our method to standardize the baseline is acceptable for the scope of this study, though not gold standard.

## Conclusion

Physically active individuals seemed to be at less risk of performing a non-good SLS. Although no difference in clinical rating between physically and non-physically active individuals emerged, we found greater knee and hip flexion kinematics in physically active individuals respect to non-active ones. The strongest predictive variable for a good clinical performance seemed to be the knee flexion. Consequently, a greater knee flexion can play a crucial role in the assessment of SLS performance and it can be viewed as an injury preventive factor.

## Additional file

Additional file 1: Supplementary material –S1. Clinical rating performances between physically active and non- physically active groups. (DOCX 14 kb)

## Abbreviations

BMI: Body Mass Index
ICC: Intraclass Correlation Coefficient
SD: Standard Deviation
sEMG: Surface Electromyography
SLS: Single Leg Squat
