# Supplementary material for: Single leg squat performance in physically and non-physically active individuals: a cross-sectional study
Source: BMC Musculoskelet Disord. 2017 Jul 14;18:299. doi: 10.1186/s12891-017-1660-8 (PMC5513318; doi:10.1186/s12891-017-1660-8)
Supplement: Supplementary file 1 — Supplementary material –S1. Clinical rating performances between physically active and non- physically active groups. (DOCX 14 kb) [file 12891_2017_1660_MOESM1_ESM.docx]

**Supplementary Material- S1.** Difference between physically active group and non- physically active group for good, fair and poor clinical rating.

| physically active group | | non-physically active group | | Sig. (2-tailed) |
| --- | --- | --- | --- | --- |
| Mean Rank | Sum of Ranks | Mean Rank | Sum of Ranks |  |
| 31.11 | 995.50 | 38.36 | 1419.50 | 0.081 |

**Legend**

CI=Confidence Interval
